# Association between sperm parameters and recurrent pregnancy loss in infertile couples: a cross-sectional study

**DOI:** 10.61622/rbgo/2026rbgo23

**Published:** 2026-05-12

**Authors:** Farzaneh Rashidi, Fatemeh Tanhaye Kalate Sabz, Nazanin Hesari, Sahar Shariatnia, Elahe Yekta, Tooba Farazmand

**Affiliations:** 1 North Khorasan University of Medical Sciences Addiction and Behavioral Sciences Research Center Bojnurd Iran Addiction and Behavioral Sciences Research Center, North Khorasan University of Medical Sciences, Bojnurd, Iran.; 2 North Khorasan University of Medical Sciences North Khorasan Faculty of Medicine Bojnurd Iran Faculty of Medicine, North Khorasan University of Medical Sciences, Bojnurd, Iran.; 3 North Khorasan University of Medical Sciences Faculty of Medicine Department of Anatomical Sciences and Pathology Bojnurd Iran Department of Anatomical Sciences and Pathology, Faculty of Medicine, North Khorasan University of Medical Sciences, Bojnurd, Iran.; 4 North Khorasan University of Medical Sciences Faculty of Medicine Department of Gynecology Bojnurd Iran Department of Gynecology, Faculty of Medicine, North Khorasan University of Medical Sciences, Bojnurd, Iran.

**Keywords:** Recurrent pregnancy loss, Sperm parameters, Risk

## Abstract

**Objective:**

Recurrent pregnancy loss (RPL) is known as a significant reproductive issue globally, which affects almost 5% of couples. Various factors contribute to the incidence of RPL. Considering the crucial role of male factors in the incidence of RPL, this study investigates the relationship between sperm parameters and RPL.

**Methods:**

This cross-sectional study was performed on 253 infertile couples who were eligible to participate and referred to the infertility center of BentolHoda Hospital from February 2022 to March 2023. The information was collected through demographic, medical, and infertility assessments, as well as laboratory examinations. Participants were categorized into two groups: couples experiencing RPL and couples facing primary or secondary infertility, which served as controls. Data were analyzed using descriptive statistical tests, the chi-square test, and logistic regression. Variations in different sperm parameters, as well as other demographic factors, were investigated between the two groups.

**Results:**

According to the findings, 32.4% of infertile couples had a history of RPL. The Chi-Square test results demonstrated a statistically significant association between motility and viscosity in couples with a history of recurrent pregnancy loss (p < 0.05). The results of the logistic regression analysis showed that the variables age (B = 0.103), drug therapy for infertility (B = -2.826), sperm viscosity (B = -1.440), and sperm motility (B = 1.113) could significantly relate to the PRL.

**Conclusion:**

The findings of this study suggest that low sperm motility, female age, and high sperm viscosity are factors that influence the occurrence of RPL.

## Introduction

Recurrent pregnancy loss (RPL) is a complication during pregnancy that is characterized by the loss of three or more pregnancies before the 20th week. The rate of prevalence varies between 1% and 5%.^(1,2)^ RPL is a complex condition affected by various elements such as hormonal imbalances, uterine structural disorders, chromosomal abnormalities, polycystic ovary syndrome, autoimmune diseases, coagulation disorders, and genetic issues, including polymorphisms. Even after addressing uterine, ovarian, and chromosomal factors, approximately 50% of cases remain idiopathic.^(3,4)^ Investigation and follow-up classically begin after three miscarriages, although diagnostic procedures may be acceptable after two miscarriages in cases involving chronic infections and advanced maternal age.^(5)^

Since males provide 50% of the embryo's genetic information, factors associated with males also influence the rates of miscarriage. Nevertheless, information regarding male factors that affect pregnancy loss is relatively scarce.^(6,7)^ Kamkar et al.^(8)^ reported increased levels of low sperm quality and sperm DNA fragmentation in the partners of women with RPL in their study. Despite this, few studies have explored the association between sperm quality and RPL.^(9)^

RPL may result from genetic factors such as the quantity and quality of sperm. During maturation, human spermatozoa is affected by environmental factors such as radiation, oxidative stress, toxic and chemical substances, and trace element deficiencies, which lead to changes in sperm karyotype, DNA, and genetic mutations that affect normal sperm function and morphology. Recent studies indicate that sperm quality, including motility, vitality, morphology, and DNA fragmentation, was significantly associated with infertility, pregnancy outcomes, and fetal health. However, findings in this area are not yet sufficient for definitive clinical guidelines.^(10-12)^

The desire for children is a significant motivator for many individuals. Failure to conceive can be a stressful experience, adversely affecting emotional well-being and social dynamics. Infertility, particularly RPL as a high-impact subset of infertility, leads to a decrease in the quality of life of infertile couples and depression.^(13,14)^ Male factors as a modifiable element in RPL management were known.^(15)^ In contrast, some studies have found conflicting results regarding specific sperm parameters, such as concentration.^(16)^

Addressing RPL can enhance couple cohesion, increase childbearing rates, and mitigate the psychological and social impact on families. Given the importance of this issue and the conflicting literature, the present study aims to investigate the relationship between RPL and sperm parameters, as well as other demographic factors.

## Methods

This cross-sectional study, employing a descriptive-analytical approach, was conducted among infertile couples referred to the infertility center of BentolHoda Hospital in Bojnurd from February 2022 to March 2023. Sampling was performed continuously, with the researcher visiting the infertility center and reviewing patient records regularly. Sample size estimation was based on Zhang et al.,^(16)^ which determined a sample of 253 couples, accounting for a 10% loss rate.

The inclusion criteria were that the couples had no known chromosomal abnormalities, and the women's ages ranged from 15 to 49 years. Exclusion criteria included hysterectomy, ovarian surgery, chemotherapy or radiotherapy, autoimmune diseases (systemic lupus erythematosus), urinary tract infections, hormone therapy in the last six months, incomplete case files, and erroneous data.

After obtaining permission from Bojnurd Medical School and the North Khorasan Province authorities, data collection began according to the entry criteria outlined in client files. The data were gathered through demographic, medical, and infertility characteristics, as well as laboratory tests. The demographic form involved the man's age, the woman's age, the woman's education level, the man's education level, the woman's occupation, the man's occupation, previous IUI, previous IVF, and Drug therapy for infertility. The laboratory form was a standard semen analysis, which usually indicates that semen volume, viscosity, sperm motility, concentration, and morphology are within acceptable ranges, suggesting good fertility potential. Specifically, a standard semen analysis includes a semen volume of at least 1.5 ml, a sperm concentration of at least 15 million sperm per ml, a total sperm motility rate of at least 40 % and at least 4 % sperm with normal morphology. The normal pH range is 7.2 to 8.0.^(17,18)^

In this analysis, sperm concentration was assessed using a sperm concentration device (Makler Concentrationing Chamber) in millions per liter, sperm motility was assessed using Computer Aided Sperm Analysis (CASA) software based on World Health Organization criteria (2010), and sperm morphology was examined using a Diff-Quick staining method.

In this research, participants (n = 253) were divided into two groups: couples with RPL (n = 85) and couples with primary infertility and secondary fertility, serving as controls (n = 155).

Following data collection, questionnaires were encoded, and data were entered into a computer. Once data entry accuracy was verified, data analysis was conducted using SPSS version 24. Descriptive statistics (central indices, dispersion, and frequency percentages), chi-square, and logistic regression were used. A confidence level of 95% (α = 5%) and a test power of 80% (β = 20%) were considered in this study.

This study is part of a research project approved by the North Khorasan University of Medical Sciences (IR Code: NKUMS.REC.1402.044). The study was performed in accordance with honesty, confidentiality, and ethics.

## Results

The study included 253 infertile couples. Abnormal sperm analysis in couples with a history of RPL showed morphology, motility, and high viscosity. Concentration was 1.97%, 3.9%, 11.46%, and 5.14 %, respectively. The characteristics of the participants are provided in table 1.

**Table 1:** Participant characteristics

Variables		Mean	Standard deviation
Age	Women	30.69	6.40
	Men	34.43	6.62
Sperm	Volume	3.72	1.69
	Concentration	56.57	33.23
	Motility	53.99	21.77
	Morphology	16.77	9.99
**Variable**		**%**	**N**
Women's education status	Elementary	46	18.2
	Middle School	52	20.6
	High School and Diploma	80	31.6
	Academic	75	29.6
Men's education status	Elementary	35	13.8
	Middle School	58	22.9
	High School and Diploma	84	33.2
	Academic	76	30
Women's job status	Employed	14	5.5
	Unemployed	14	5.5
	Freelance	225	88.9
Man's job status	Employed	24	9.5
	Unemployed	228	90.1
	Freelance	1	0.4
Viscosity	Less than high	1.97	5
	Normal	76.29	193
	High	21.74	55
History of recurrent pregnancy loss	Yes	32.4	82
	No	67.6	171
Previous IVF	Yes	10	4
	No	243	96
Previous IUI	Yes	34	13.4
	No	219	86.6
Drug therapy for infertility	Yes	102	40.3
	No	151	59.7

The results of Chi-Square test showed a statistically significant association between Motility and Viscosity in couples both with and without a history of RPL (p<0.05). The Chi-Square test analyses revealed no statistically significant association in concentration, volume, and morphology between couples with and without a history of RPL (p>0.05) (Table 2).

**Table 2:** Comparison of sperm parameters in couples with and without recurrent pregnancy loss

Recurrent pregnancy loss		Yes	No	p-value (Chi-Square)
Normal morphology sperm				
	Yes	85(33.59)	155(61.27)	0.629
	No	5(1.97)	8(3.17)	
Sperm viscosity				
	Low	4(1.58)	1(0.39)	0.0001
	Normal	59(23.32)	135(53.36)	
	High	29(11.46)	25(9.89)	
Normal sperm motility				
	Yes	80(31.62)	115(45.45)	0.004
	No	10(3.95)	48(18.97)	
Normal sperm volume				
	Yes	88(34.79)	154(60.86)	0.303
	No	2(0.79)	9(3.56)	
Normal sperm concentration				
	Yes	77(30.44)	153(60.47)	0.230
	No	13(5.14)	10(3.95)	

The results of logistic regression showed that the variables age (B=0.103), drug therapy for infertility (B= -2.826), sperm viscosity (B= -1.440), and sperm motility (B=1.113) could significantly predict the variance of recurrent pregnancy loss (Table 3).

**Table 3:** Association of sperm parameters with RPL based on logistic regression test

	B	S.E.	df	Sig.	Exp(B)
Female age	0.103	0.045	1	0.022	1.108
Male age	-0.019	0.040	1	0.641	0.981
Drug therapy for infertility	-2.826	0.544	1	0.000	0.059
Previous IUI	0.737	0.759	1	0.331	2.090
Previous IVF	0.839	1.018	1	0.410	2.314
Viscosity sperm(High)	-1.440	0.419	1	0.001	0.237
Morphology sperm	-0.319	0.932	1	0.732	0.727
Sperm motility	1.113	0.494	1	0.024	3.044
Sperm volume	1.452	0.932	1	0.119	4.270
Sperm concentration	0.065	1.044	1	0.950	1.067
Constant	-5.888	2.824	1	0.037	0.003

S.E: Standard error; df: Degree of freedon; Exp: Exponentiated coefficient

## Discussion

This cross-sectional, descriptive-analytical study investigated the relationship between sperm parameters and other demographic factors, as well as PRL. The findings of this research suggest that maternal age plays a crucial role concerning RPL. Similarly, the findings of Carlini et al.^(19)^ It also demonstrated a significant association between the participants’ age and the occurrence of RPL, indicating that the likelihood of RPL increases with age. This finding is consistent with the results of the present study. With increasing age, both sperm quality and egg quality, as well as related parameters, decline, leading to a reduced chance of successful fertilization and an increased risk of miscarriage.^(19)^ Additionally, Cao et al.^(20)^ identified advancing age as one of the most significant causes of RPL. To mitigate this issue, an earlier pregnancy can be considered a potential way to reduce the occurrence of RPL.

Sperm morphology was another factor examined in our study, and the findings indicated no considerable connection between sperm morphology and RPL. This aligns with the findings of Gil-Villa et al.,^(21)^ who indicated that sperm morphology alone cannot be considered a determining factor in RPL. However, studies by Ahmadi et al.^(3)^ and Eisenberg et al.^(4)^ indicate that abnormal sperm morphology is associated with early embryo mortality and that defective DNA in sperm negatively affects the satisfactory expression of paternal genes during early embryonic development.^(3,4)^

Sperm concentration was another factor considered in our study, and the results showed no significant association between this factor and RPL. Several other studies have confirmed our results and found that sperm concentration alone does not affect the occurrence of RPL. They indicate that while sperm concentration may influence other factors, such as motility, it does not lead to RPL.^(7)^

This study also examined semen volume as a factor, and no significant association was found between this factor and RPL. In alignment with this result, Li et al.^(9)^ also reported that semen volume has no effect on miscarriage and, like sperm concentration, is not effective on its own. On the other hand, Carlini et al. (2017)^(19)^ presented a contrasting view, suggesting that semen volume alone could contribute to RPL by limiting sperm motility.

Sperm motility was another factor considered in our study, and the results indicated a significant relationship between this factor and RPL. However, the results of various studies did not demonstrate a significant correlation that would identify sperm motility as an independent factor related to RPL.^(12,21)^

Based on the available research, the association between sperm motility and recurrent pregnancy loss (RPL) remains inconclusive. While these studies suggest a significant relationship, the current findings from several studies indicate that there is no statistically significant association between sperm motility and RPL. In a survey of 1,485 participants, no statistically significant alterations in sperm parameters (including motility) were found between men from couples with RPL and controls.^(22)^ Also, in a case-control study comparing men from couples with RPL with controls, no significant differences in sperm motility or other standard semen parameters were found.^(23)^ One reason for the differing results is that different studies suggest that this factor is age-dependent and does not have an independent effect on RPL. From the researchers’ perspective, race and other environmental factors that affect sperm quality could help elucidate the discrepancies between our study's findings and those of different studies.

One important factor in sperm quality is intact sperm DNA; however, this study did not evaluate it. A multicentre study presented a positive association between sperm DNA damage and the occurrence of RPL, highlighting the importance of examining sperm parameters when diagnosing the causes of RPL.^(24)^ In addition to sperm parameters, various studies on multiple systemic and local factors—such as celiac disease, metabolic disorders, immune factors, vitamin D, and viral infections—have contributed to understanding the occurrence of RPL.^(25-29)^ One of the relevant tests for RPL is cell-free DNA testing, and studies have shown that these tests are important for identifying fetal chromosomal abnormalities and can be helpful in the clinical management of patients with RPL.^(30,31)^ Consideration of uterine factors and their structure using methods such as 3D ultrasound may also be important in the evaluation of patients.^(32)^ They also found that women with unexplained RPL have a longer time to become pregnant than women without a history of miscarriage, indicating possible fertility problems in this group.^(33)^ A recent study suggests that targeted therapies, such as corticosteroids, can improve pregnancy outcomes in women with a history of RPL.^(34)^

## Conclusion

This investigation demonstrated that factors such as women's age, sperm motility, and high viscosity are among the most significant factors influencing RPL. Paying attention to factors that could reduce or eliminate the effects of these two elements may help reduce RPL. Key strategies include reducing the age of pregnancy and addressing social and environmental factors that discourage couples from conceiving at a younger age. We hope that the findings of this study will serve as a foundation for future research on the factors affecting recurrent pregnancy loss, thereby contributing to a reduction in its incidence and promoting fertility

## Data Availability

The research data are described in the article presented.
